# Stigma Impacts Health Disparities and Inequities in LGBTQ and People of Color Aging With HIV

**DOI:** 10.1093/geroni/igab046.848

**Published:** 2021-12-17

**Authors:** Anna Egbert, Paul Nash, Mark Brennan-Ing, Stephen Karpiak

**Affiliations:** 1 Ronin Institute, Montclair, New Jersey, United States; 2 University of Southern California, Los Angeles, California, United States; 3 Brookdale Center for Healthy Aging Hunter College, CUNY, New York, New York, United States; 4 GMHC, National Resource Center on Aging and HIV, New York, New York, United States

## Abstract

The impact of stigmatizing attitudes and discriminatory behaviors on health disparities and inequities in non-heterosexual individuals, people of color (PoC), older adults, and persons living with HIV becomes increasingly recognized. This quartette of stigmatized characteristics elevates the risk of barriers to medical services, burden of disease and unfavorable health outcomes in LGBTQ-PoC aging with HIV. Using data from ROAH 2.0 study (N=723), we explored facets of stigma, barriers to medical services and health status in racial/ethnic minorities of older adults with HIV (OAH) living in California, New York, and Illinois. Stigma was evident in >50% of OAH who expressed reservation to self-disclose HIV status. Importantly, 20%-24% of Asian, Black/African-American, Hispanic/Latinx and Multiracial vs. 7% White OAH withheld this information from at least one health care provider. Over 10% of OAH experienced prejudice/discrimination while accessing service. Non-disclosure and prejudice/discrimination were linked to lower self-rated health status, thus, evidencing stigma-related health burden.

